# Supplementary material for: Academic User View: Organ-on-a-Chip Technology
Source: Biosensors (Basel). 2022 Feb 16;12(2):126. doi: 10.3390/bios12020126 (PMC8869899; doi:10.3390/bios12020126)
Supplement: Supplementary file 1 [file biosensors-12-00126-s001.zip › Suppl-Figures.pdf]

Supplementary Material

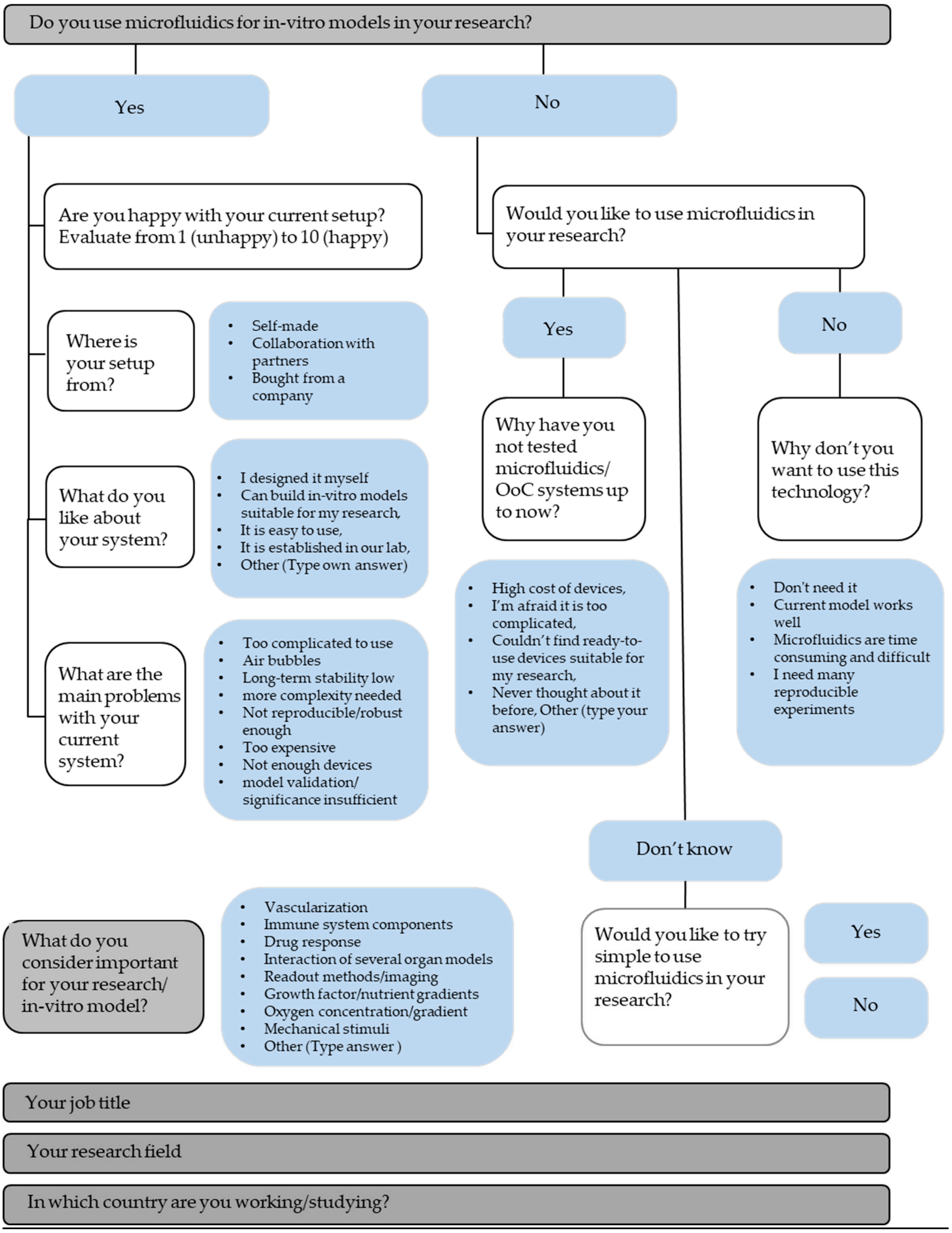

Figure S1. Survey scheme used for this paper.

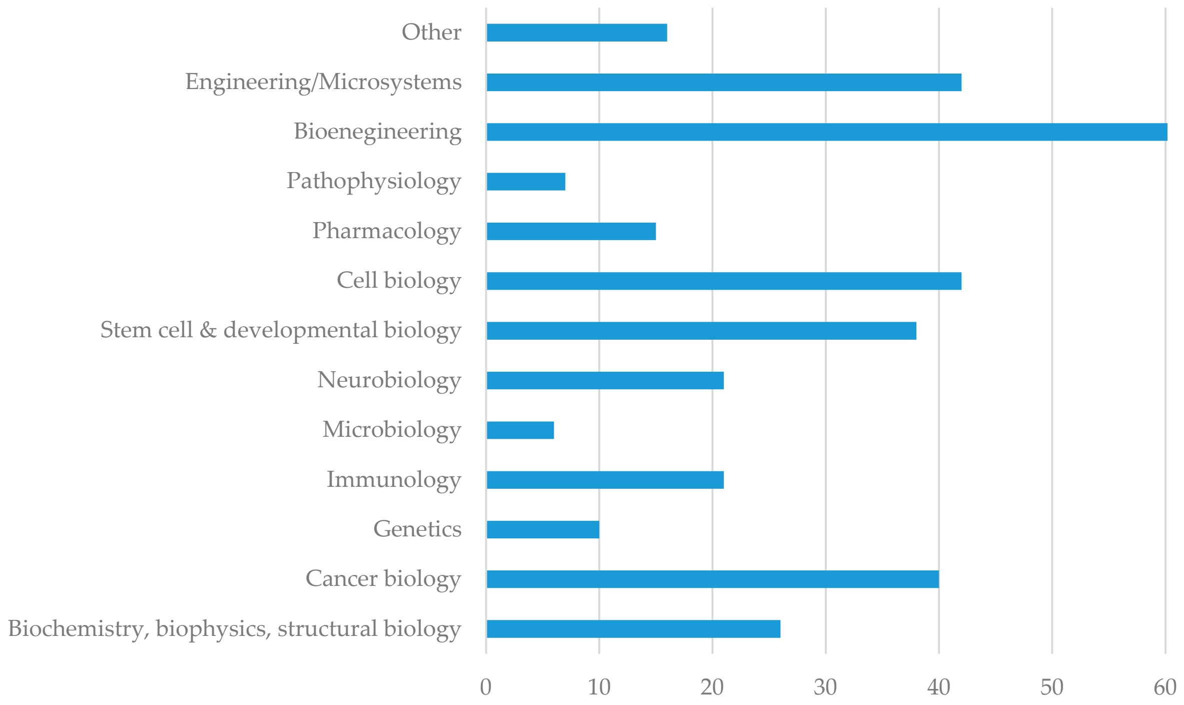

Figure S2. Survey participants by research field (multiple options).

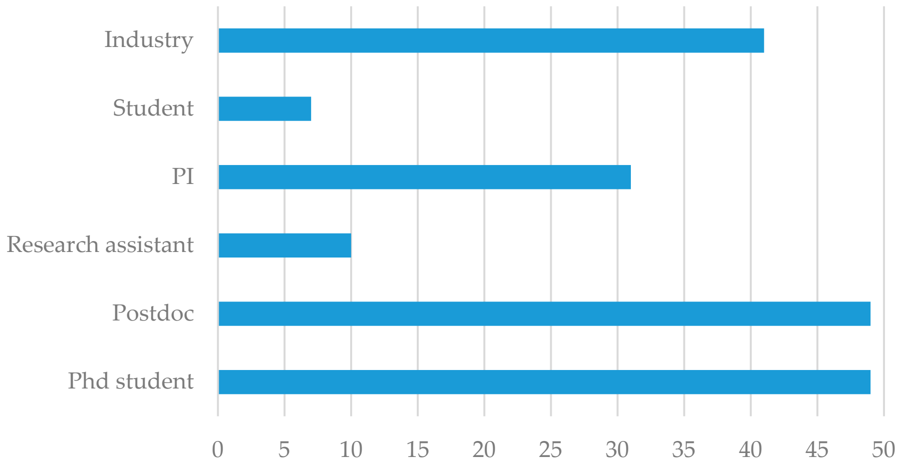

Figure S3. Survey participants by position.

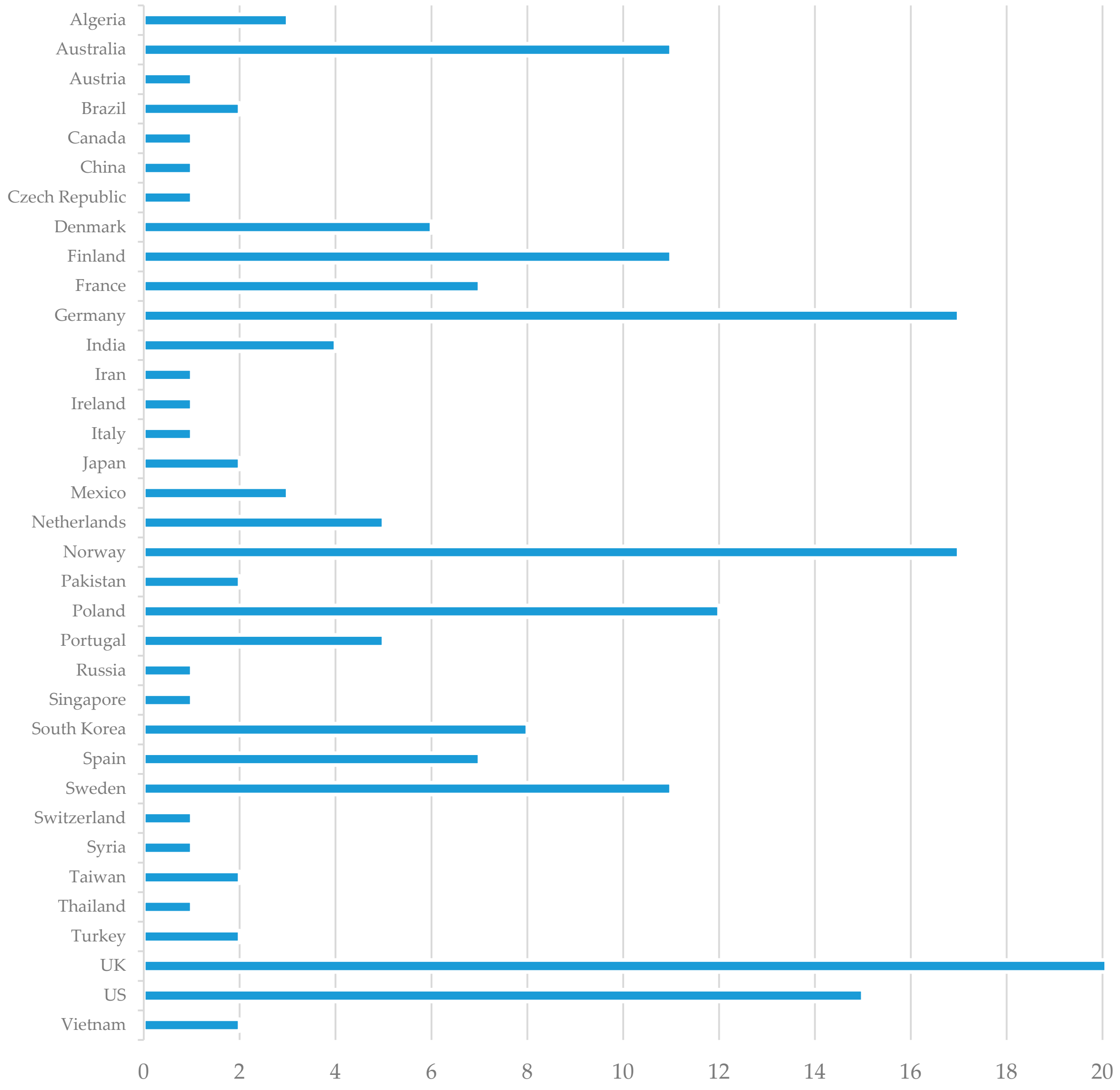

Figure S4. Survey participants by country.

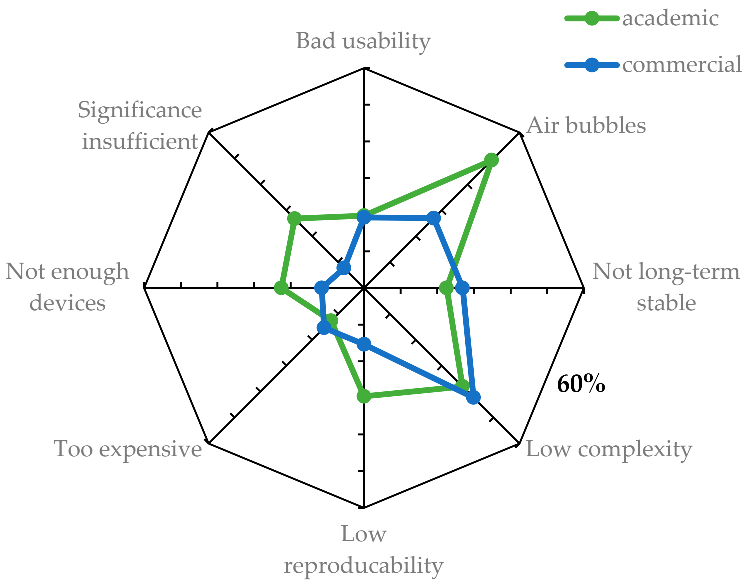

Figure S5. User feedback on problems with OoC devices by background (industry vs academia).

Table S2: List of asked institutions and Social network groups

Directly mailed universities/departments:
Institute of Basic Medical Science, University of Oslo, Norway
Institute of Human Genetics, Polish Academy of Science, Poznan, Poland
School of Biomedical Engineering, The University of Sydney, Australia
Faculty of Engineering, Department of Materials, Imperial College London, UK
School of Chemistry and Bioscience, University of Bradford, UK
University of South Australia, UniSA Cancer Research Institute & Future Industries Institute, Australia
John Hopkins University, Baltimore, USA
KTH Royal Institute of Technology, Micro- and Nanosystems, Gothenburg, Sweden
Tampere University, Finland
University of Melbourne, Australia
University of Wyoming, USA
University of Tubingen, Germany

Networks addressed:
“Nordic Organ on Chip Society” (Finland, Denmark, Sweden, Norway and Iceland)
“Organ-on-a-Chip technology” Facebook group
“Organs-on-chips” & “3D cell biology” groups on LinkedIn
